# Donation type and the effect of pre-transplant donor specific antibodies – Data from the Swiss Transplant Cohort Study

**DOI:** 10.3389/fimmu.2023.1104371

**Published:** 2023-02-15

**Authors:** Olivier de Rougemont, Yun Deng, Lukas Frischknecht, Caroline Wehmeier, Jean Villard, Sylvie Ferrari-Lacraz, Déla Golshayan, Monique Gannagé, Isabelle Binet, Urs Wirthmueller, Daniel Sidler, Thomas Schachtner, Stefan Schaub, Jakob Nilsson, the Swiss Transplant Cohort Study, Patrizia Amico

**Affiliations:** ^1^ Department of Surgery and Transplantation, University Hospital Zurich, Zurich, Switzerland; ^2^ Department of Immunology, University Hospital Zurich (USZ), Zurich, Switzerland; ^3^ Clinic for Transplantation Immunology and Nephrology, University Hospital Basel, Basel, Switzerland; ^4^ Transplantation Immunology Unit and National Reference Laboratory for Histocompatibility, Department of Diagnostic, Geneva University Hospitals, Geneva, Switzerland; ^5^ Transplantation Center, Lausanne University Hospital, Lausanne, Switzerland; ^6^ Service of Immunology and Allergy, Lausanne University Hospital, University of Lausanne, Lausanne, Switzerland; ^7^ Nephrology & Transplantation Medicine, Cantonal Hospital St. Gallen, St. Gallen, Switzerland; ^8^ Department of Laboratory Medicine, Inselspital, Bern University Hospital and University of Bern, Bern, Switzerland; ^9^ Department of Nephrology and Hypertension, Inselspital, Berne University Hospital and University of Berne, Berne, Switzerland; ^10^ Division of Nephrology, University Hospital Zurich, Zurich, Switzerland

**Keywords:** kidney transplantation, donor specific antibodies, ABMR, graft loss, virtual cross-match, living donation, DBD, DCD

## Abstract

**Introduction:**

The type of donation may affect how susceptible a donor kidney is to injury from pre-existing alloimmunity. Many centers are, therefore, reluctant to perform donor specific antibody (DSA) positive transplantations in the setting of donation after circulatory death (DCD). There are, however, no large studies comparing the impact of pre-transplant DSA stratified on donation type in a cohort with a complete virtual cross-match and long-term follow-up of transplant outcome.

**Methods:**

We investigated the effect of pre-transplant DSA on the risk of rejection, graft loss, and the rate of eGFR decline in 1282 donation after brain death (DBD) transplants and compared it to 130 (DCD) and 803 living donor (LD) transplants.

**Results:**

There was a significant worse outcome associated with pre-transplant DSA in all of the studied donation types. DSA directed against Class II HLA antigens as well as a high cumulative mean fluorescent intensity (MFI) of the detected DSA showed the strongest association with worse transplant outcome. We could not detect a significant additive negative effect of DSA in DCD transplantations in our cohort. Conversely, DSA positive DCD transplants appeared to have a slightly better outcome, possibly in part due to the lower mean fluorescent intensity (MFI) of the pre-transplant DSA. Indeed when DCD transplants were compared to DBD transplants with similar MFI (<6.5k), graft survival was not significantly different.

**Discussion:**

Our results suggest that the negative impact of pre-transplant DSA on graft outcome could be similar between all donation types. This suggests that immunological risk assessment could be performed in a similar way regardless of the type of donor kidney transplantation.

## Introduction

Antibody mediated rejection (ABMR) is the main cause for graft loss at later time points after kidney transplantation (1–3). The presence of donor specific antibodies (DSA) that are specific for non-self human leukocyte antigen (HLA) variants present on cells in the donor graft has emerged as the main risk factor for ABMR development (4). Several previous studies have shown an increased risk for ABMR and graft loss in recipients where DSA are detected pre-transplantation and anti-HLA antibodies are therefore continuously monitored in patients on the kidney transplant waiting list (4–9). It is possible that the type of kidney donation could affect the impact of pre-transplant DSA based in part of on differences in cold and warm ischemia time. A recent large study found a larger hazard ratio for graft loss associated with living donor (LD) kidney transplantation as compared to deceased donor transplantation wheras a previous large study showed data suggesting a more detrimental effect in the deceased donor setting (5, 10). Both of these studies did not find a significant difference in outcome if the pre-transplant DSA were directed against HLA Class I or Class II antigens, which is in contrast to our own previously published data that showed an increased risk in the setting of DSA directed at Class II antigens (8). To our knowledge, there has however been no previous large study with an extended follow-up time examining the impact of pre-transplant DSA, stratified on donation type, on transplant outcome in the setting of a complete virtual cross-match (vXM) where donor HLA typing data is available for all detected anti-HLA antibodies. The impact of pre-transplant DSA on the outcome of kidney transplantation in the setting of donation after circulatory death (DCD) is of particular interest. Initially, DCD transplantations showed an inferior outcome as compared to transplantations performed in the setting of donation after brain death (DBD), but significant improvements during the last 10 years has led to similar outcomes between DBD and DCD transplants in many centers (11, 12). In Switzerland DCD donation was reintroduced in 2011, and in 2017 DCD donors made up 27% of all deceased donors (13). Despite these improvements, there is still uncertainty on how the extended warm ischemia time in the setting of DCD transplants may potentially affect the susceptibility of the graft to pre-existing alloimmunity. Previous studies also showed an inferior graft survival for DCD grafts in the setting of re-transplantation, which could potentially be due to increased sensibility to pre-existing alloimmunity (14). Theoretically, extended warm ischemia time with tissue and cell hypoxia could be postulated to result in an increase in tissue inflammation and endothelial activation with subsequent upregulation of HLA expression, which might predispose the graft to pre-existing alloimmunity (15). Given these uncertainties, many centers are reluctant to accept DCD organ offers for patients with pre-transplant donor specific antibodies (DSA) even if the measured mean fluorescence intensity (MFI) of the detected DSA is low. With increasing numbers of DCD donors, this further limits the possibilities of transplanting highly immunized recipients and increases waiting times for immunized recipients (16). To our knowledge, there are no reports in the literature on the outcome of transplantation in DSA positive DCD transplants. In order to improve pre-transplant immunological risk assessment in the setting of kidney transplantation, we investigated the impact of pre-transplant DSA on the outcome of kidney transplantation in relation to the type of donation. We evaluated the incidence of biopsy proven antibody mediated rejection (ABMR), decline in graft function as measured by estimated glomerular filtration rate (eGFR) slope and death censored graft loss in a cohort of 1282 DBD transplants, 130 DCD transplantations as well as 803 LD transplants included in the Swiss Transplant Cohort Study (STCS).

## Methods

### Study design and STCS cohort description

The Swiss Transplant Cohort Study (STCS, www.stcs.ch) is a nationwide cohort study recruiting patients with solid organ transplantation at all six transplantation centers in Switzerland (Basel, Bern, Geneva, Lausanne, St Gallen, and Zurich). The current study (project number FUP144) was nested within the STCS. The Cantonal Ethics Committee of Zurich (BASEC-Nr.2021-0083) separately approved this sub-study.

Patients who received kidney transplants between May 2008 and December 2017 in Switzerland were recruited into the study. With a total of 2657 kidney transplants in the database, 2215 transplants (n=2179 patients) were included for the final analysis. For our specific study, the following exclusion criteria were applied: 1) the baseline data prior to transplantation was missing (n=10), 2) patients with multi-organ transplantation (n=158), 3) patients under the age of 18 (n=90), 4) transplants with incomplete virtual crossmatch (n=28), 5) loss of observation after first year of follow-up (n=3). Furthermore, ABO-incompatible living donor transplants (n=153) were excluded from the analysis.

The baseline data, such as the donor type and routine laboratory data were collected at the time of transplantation. Follow-up data on transplant outcome was collected at 6 months after transplantation, and then annually with results entered into the STCS database.

### HLA typing and detection of HLA antibodies

HLA typing was performed by DNA based HLA-typing technology using blood samples. Either sequence-specific oligonucleotide (SSO) or sequence-specific primer (SSP) technologies were used. The majority (n=2207, 99.6%) of class I and class II HLA antibody screeening was done using a Luminex bead-based platform, while a few were screened by ELISA (n=8, 0.4%). A total of 1591 (72%) of the patients were analyzed by Luminex single-antigen bead (SAB) technology (LABScreen Single Antigen; OneLambda) to detect the presence of pre-transplant anti-HLA antibodies. This was done either directly or after a positive screening test with the mixed bead analysis (LABScreen Mixed, OneLambda). In total 27% of the patients (n= 616) were only analyzed with a mixed bead analysis that was negative and received no subsequent SAB analysis. A complete virtual cross-match (vXM) was performed for all patients by comparing the profile of the donors’ HLA typing results with the specificities of the recipients’ anti-HLA antibodies. If the patient had anti-HLA antibodies directed at a locus that had not been previously typed in the donor, additional donor typing was performed. Both historical and current HLA antibodies with a mean fluorescence intensity (MFI) > 500–1000 (depending on the center-specific cutoff) were included, with the majority of centers reporting antibodies >500 MFI. Cumulative DSA MFI was calculated by adding the MFIs of all detected DSA at HLA antigen resolution.

### Definitions of rejection and graft loss

The diagnosis of transplant rejection was dependent on the assessment of renal allograft biopsies. Biopsy specimens were collected and analyzed based on the local protocol at each transplantation center by specialized pathologists. Details on the biopsy evaluation were documented either by the individual Banff scores or as descriptive text that was later translated and graded according to the Banff 2017 criteria and classified as ABMR (capillaritis, glomerulitis, C4d positivity) and TCMR (interstitial inflammation, tubulitis) (17). Biopsies with findings of “borderline changes” and “C4d positive staining without evidence of rejection” were not considered as rejection in our study (18). Graft loss was defined as return to dialysis after transplantation or re-transplantation without prior dialysis. Death censored graft loss was considered as the main investigative outcome after transplantation.

### Estimation of eGFR and eGFR slope

We estimated renal allograft function in the form of estimated glomerular filtration rate (eGFR) by using the Chronic Kidney Disease Epidemiology Collaboration (CKD-EPI) 2009 creatinine equation (19). In our cohort, 69 patients who had eGFR values exceeding the normal diagnostic range (eGFR >150 mL/min/1.73 m^2^) were interpreted as wrongfully entered and were excluded from the study (the database contains approximately 23 000 creatinine values).

The rate of eGFR decline was defined as eGFR slope and presented in units of “ml/min/1.73m^2^/year”. It was calculated using the eGFR value at year 1 after transplantation as the baseline value. In the longitudinal analysis (defined as “eGFR mean annual slope”), the absolute eGFR slope value was illustrated in each follow-up year in the respective groups. In order to catch the latest follow-up time before either loss of graft or end of the observation period from each individual patient, the “eGFR mean total slope” was summarized using the last recorded eGFR slope value in the respective groups.

### Data processing and statistical analysis

All the pre-processing of the raw data exported from STCS database and the subsequent statistical analyses were carried out in R programming environment (R, version 4.0.3 and RStudio, version 1.3.1093). Various packages used in the study included “dplyr” (1.0.7), “ggplot2” (3.3.6), “lubridate” (1.8.0), “pacman” (0.5.1), “rio” (0.5.29), “stats” (4.0.3), “survminer” (0.4.9), tibble” (3.1.6) and “tidyr” (1.1.4). A log-rank test was applied to compare the Kaplan-Meier survival plots. A Mann-Whitney U test to was used to analyze unpaired data with a non-Gaussian distribution for 2 groups’ comparison. While one-way analysis of variance (ANOVA), followed by Dunn’s *post hoc* test was used for comparisons of more than 2 groups. The longitudinal study was analyzed using two-way ANOVA, followed by Tukey’s multiple comparisons. Hazard ratio (HR), the corresponding 95% confidence interval (CI) and p values for different variables were obtained by univariate Cox proportional hazards regression analysis. To perform the multivariate analysis and overcome the multi-collinearity between several of the studied variables, a partial least squares (PLS) regression was used to model the dependence relationship between one dependent outcome variable and multiple independent variables in an exploratory fashion.

## Results

### Study population characteristics

**Figure 1:**
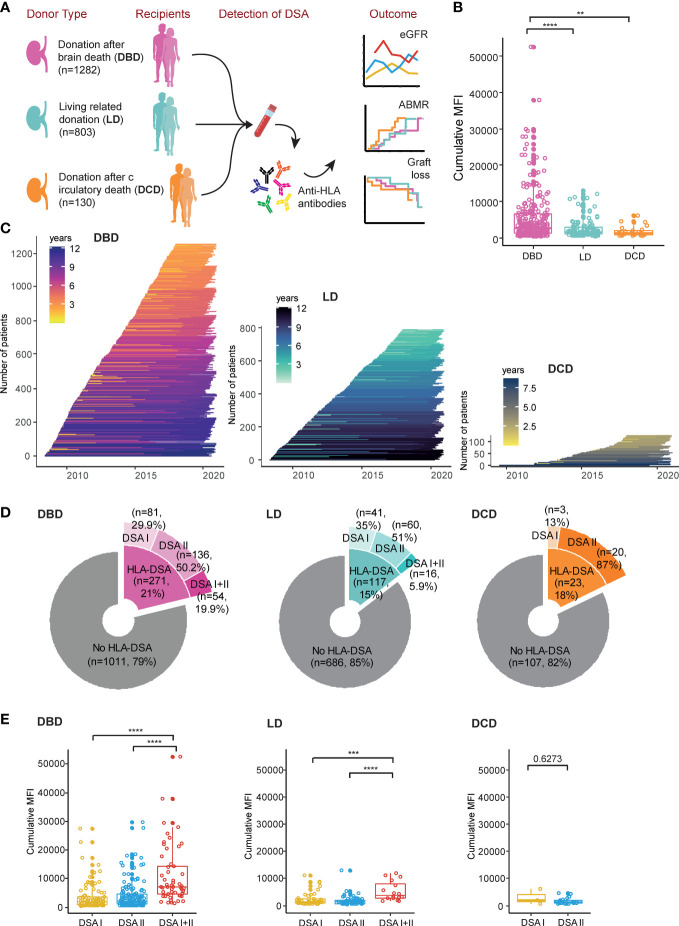
Overview of the STCT cohort and the grouping of patients based on donation type. **(A)** Flowchart indicating the number of patients for the different donation types (DBD, LD and DCD), detection of DSA and the major analyses performed in this study. **(B)** The distribution of the cumulative MFI value of DSA positive patients. **(C)** Follow-up time in each individual patient. **(D)** Details of the patients in the “no HLA-DSA” group and different DSA sub-groups. **(E)** The summary of the cumulative MFI value of different DSA classes in the different donation types. **p<0.01, ***p<0.001, ****p<0.0001.

In total 2215 kidney transplantations performed in Switzerland between 2008 and 2017 were included in the study. An overview of the included patients stratified on donation type as well as an overview of the follow-up time is shown in **Figures 1A, C** as well as in **Table 1**. The cumulative MFI in DSA positive transplants was significantly higher in DBD recipients as compared to both DCD and LD recipients (**Figure 1B**). The fraction of transplants that were performed in the setting of a pre-transplant DSA was however comparable between the different types of donation (DBD 21%, LD 15% and DCD 18%) (**Figure 1D**). Most DSA positive patients had DSA directed at Class II HLA antigens (**Figure 1D**) and patients with DSA directed at both Class I and Class II had significantly higher cumulative MFI than patients with DSA directed at either Class alone (**Figure 1E**).

**Table 1:** Characteristics of the recipient transplanted with kidney from different donor types with and without pre-transplant DSA.

DBD			
Characteristics	DSA	No DSA	p value (DSA vs. No DSA)
Number of patients	271 (21.1%)	1011 (78.9%)	
Age at transplantation (mean value)	52.56	55.44	0.58
Female gender (Reipient)	126 (44%)	355 (35%)	<0.0001
Age (Donor) (mean vlaue)	50.67	51.75	0.0006
Female gender (Donor)	120 (44.3%)	447 (44.4%)	0.949
Previously transplanted	149 (55%)	137 (14%)	<0.0001
Previous pregnancy (Female only)	30 (24%)	82 (23%)	0.39
Previous blood transfusion	149 (55%)	303 (30%)	<0.0001
Immunosuppression			<0.0001
FK-MPA-Pred	231 (85%)	729 (72%)	
CyA-MPA-Pred	33 (12%)	231 (23%)	
CNI-based other	6 (2%)	16 (2%)	
mTOR-containing	1 (0%)	26 (3%)	
Other	0 (0%)	7 (1%)	
Induction therapy			<0.0001
ATG/Thymo+/- lvlg	176 (65%)	138 (14%)	
Basiliximab	94 (35%)	850 (84%)	
None	1 (0%)	23 (2%)	
Underlying renal disease			<0.0001
Glomerulonephritis	59 (21.8%)	216 (21.4%)	
ADPKD	42 (15.5%)	194 (19.2%)	
Diabetic nephropathy	18 (6.6%)	97 (9.6%)	
Vascular nephropathy	22 (8.1%)	130 (12.9%)	
Interstitial nephropathy	7 (2.6%)	38 (3.8%)	
Other			
Not specified	40 (14.8%)	124 (12.3%)	
Reflux/Pyelonephritis	10 (3.7%)	48 (4.7%)	
Hereditary (not ADPKD)	5 (1.8%)	29 (2.9%)	
Congenital	9 (3.3%)	17 (1.7%)	
Unknown	59 (21.8%)	118 (11.7%)	
HLA mismatch			
A, % with 0/1/2	14.4/46.5/39.1	14.4/42.9/42.6	0.322
B, % with 0/1/2	6.6/41.3/52	6.4/38.2/55.4	0.862
DRB1, % with 0/1/2	11.8/56.8/31.4	17.2/54.9/27.9	0.827
Cold ischemia time (DD)/h (mean value)	9.93	10.31	0.817
DCD			
Characteristics	DSA	No DSA	p value (DSA vs. No DSA)
Number of patients	23 (17.7%)	107 (82.3%)	
Age (mean value)	50.7	53.67	0.17
Female gender	10 (43%)	43 (40%)	0.62
Age (Donor) (mean vlaue)	45.61	52.01	0.41
Female gender (Donor)	5 (21.7%)	45 (42.1%)	<0.0001
Previously transplanted	6 (26%)	5%	0.004
Previous pregnancy (Female only)	2 (20%)	5 (12%)	0.22
Previous blood transfusion	13 (57%)	27 (25%)	<0.0001
Immunosuppression			0.86
FK-MPA-Pred	22 (96%)	97 (91%)	
CyA-MPA-Pred	1 (4%)	9 (8%)	
CNI-based other	0 (0%)	1 (1%)	
mTOR-containing	0 (0%)	0 (0%)	
Other			
Induction therapy			0.391
ATG/Thymo+/- lvlg	20 (87%)	74 (69%)	
Basiliximab	3 (13%)	32 (30%)	
None	0 (0%)	1 (1%)	
Underlying renal disease			0.183
Glomerulonephritis	2 (8.7%)	25 (23.4%)	
ADPKD	2 (8.7%)	23 (21.5%)	
Diabetic nephropathy	2 (8.7%)	13 (12.1%)	
Vascular nephropathy	1 (4.3%)	14 (14.1%)	
Interstitial nephropathy	6 (26.1%)	1 (0.9%)	
Other			
Not specified	1 (4.3%)	14 (13.1%)	
Reflux/Pyelonephritis	/	3 (2.8%)	
Hereditary (not ADPKD)	1 (4.3%)	1 (0.9%)	
Congenital	/	4 (3.7%)	
Unknown	9 (39.1%)	9 (8.4%)	
HLA mismatch			
A, % with 0/1/2	13.0/47.8/39.1	5.6/49.5/44.9	0.573
B, % with 0/1/2	4.3/26.1/69.6	4.7/32.7/62.6	0.464
DRB1, % with 0/1/2	8.7/52.2/39.1	15.0/57.0/28.0	0.539
Cold ischemia time (DD)/h (mean value)	9.16	9.08	0.186
LD			
Characteristics	DSA	No DSA	p value (DSA vs. No DSA)
Number of patients	117 (14.6%)	686 (85.45%)	
Age at transplantation (mean value)	50	49	0.003
Female gender (Reipient)	61 (55%)	211 (31%)	<0.0001
Age (Donor) (mean vlaue)	53.62	53.7	0.64
Female gender (Donor)	65 (55.6%)	442 (64.43%)	0.006
Previously transplanted	26 (22%)	79 (12%)	<0.0001
Previous pregnancy (Female only)	13 (21%)	43 (20%)	0.86
Previous blood transfusion	42 (36%)	149 (22%)	<0.0001
Immunosuppression			0.003
FK-MPA-Pred	101 (86%)	538 (78%)	
CyA-MPA-Pred	10 (9%)	117 (17%)	
CNI-based other	4 (3%)	3 (0%)	
mTOR-containing	1 (1%)	22 (3%)	
Other	1 (1%)	6 (1%)	
Induction therapy			<0.0001
ATG/Thymo+/- lvlg	79 (68%)	62 (9%)	
Basiliximab	38 (32%)	591 (86%)	
None	0 (0%)	33 (5%)	
Underlying renal disease			0.014
Glomerulonephritis	28 (23.9%)	209 (30.5%)	
ADPKD	31 (26.5%)	124 (18.1%)	
Diabetic nephropathy	7 (6.0%)	47 (6.9%)	
Vascular nephropathy	8 (6.8%)	72 (10.5%)	
Interstitial nephropathy	2 (1.7%)	25 (3.6%)	
Other			
Not specified	9 (7.7%)	74 (10.8%)	
Reflux/Pyelonephritis	8 (6.8%)	40 (5.8%)	
Hereditary (not ADPKD)	5 (4.3%)	27 (3.9%)	
Congenital	4 (3.4%)	21 (3.1%)	
Unknown	15 (12.8%)	47 (6.9%)	
HLA mismatch			
A, % with 0/1/2	13.7/47.0/39.3	18.4/50.6/31.0	0.311
B, % with 0/1/2	6.0/42.7/51.3	13.8/44.3/41.8	0.204
DRB1, % with 0/1/2	7.7/53.8/38.5	18.2/50.4/31.3	0.769
Cold ischemia time (DD)/h (mean value)	2.29	1.74	0.003

### DCD transplantations show similar outcomes

**Figure 2:**
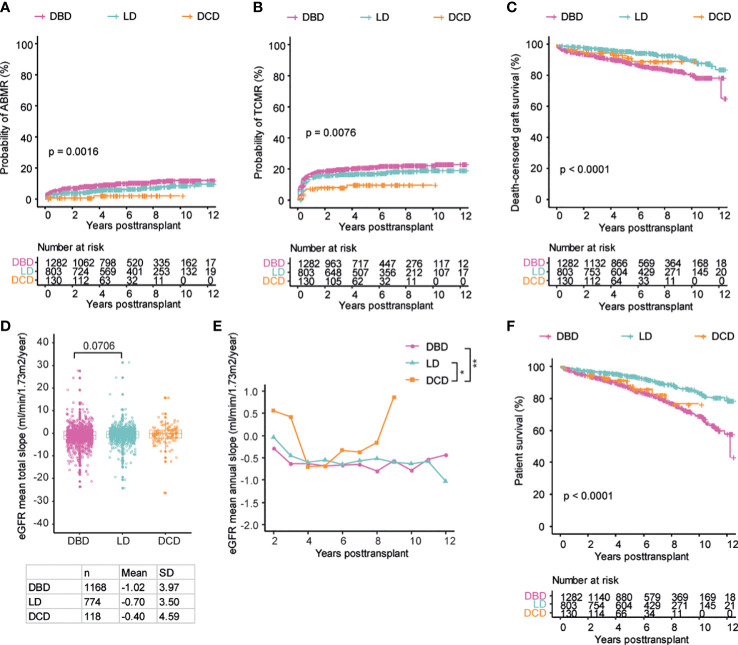
Patients receiving DCD transplants show similar outcomes as the DBD and LD patients regarding the development of ABMR, TCMR, graft survival and graft function. Cumulative incidence of ABMR **(A)**, TCMR **(B)**, death-censored graft survival **(C)**, the total mean slope of eGFR **(D)**, the collective longitudinal mean annual slope of eGFR **(E)**, and overall patient survival **(F)** in patients with the three different donation types respectively. Log-rank test was used to test p value of the Kaplan-Meier survival curves for **(A–C)** and **(F)**. One-way ANOVA analysis with Dunn’s *post hoc* test was used for **(D)** and two-way ANOVA analysis with Tukey’s multiple comparisons as a *post hoc* test was used for **(E)** to assess p values; *p<0.05, **p<0.01.

In order to investigate if the type of donation affected the outcome of transplantation, we compared DBD, LD and DCD transplants with regards to ABMR, TCMR, death censored graft survival and decline in graft function as measured by eGFR slope. Patients receiving a DCD transplant had significantly reduced incidence of both AMBR and TCMR both as compared to DBD and LD transplants (**Figures 2A, B**). LD transplants displayed a significantly higher graft survival as compared to DBD transplants with DCD transplants showing an intermediate graft survival (**Figure 2C**). Decline in graft function was greatest among recipients of a DBD graft whereas DCD transplants appeared to have the best preservation of kidney function both when annual and total eGFR slopes were calculated (**Figures 2D, E**). As expected, recipients of LD grafts had the best overall survival whereas DBD and DCD transplants showed similar overall survival (**Figure 2F**). DCD transplant recipients in general received more intensive induction therapy as well as CNI based maintenance immunosuppression (**Table 1**). In order to investigate how this affected rejection and graft loss we performed a subgroup analysis on DBD and DCD transplants that were made with ATG induction therapy and that did not receive Cyclosporin A based maintenance immunosuppression. Our results within this subgroup were similar to the results in the whole cohort concerning the risk of ABMR, TCMR and graft loss (**Supplementary Figures 1A–C**). In summary, we found evidence of lower rates of ABMR and TCMR as well as better preserved graft function in DCD transplants and similar overall survival in DCD and DBD recipients.

### Pre-transplant DSA are associated with inferior outcome regardless of donation type

**Figure 3:**
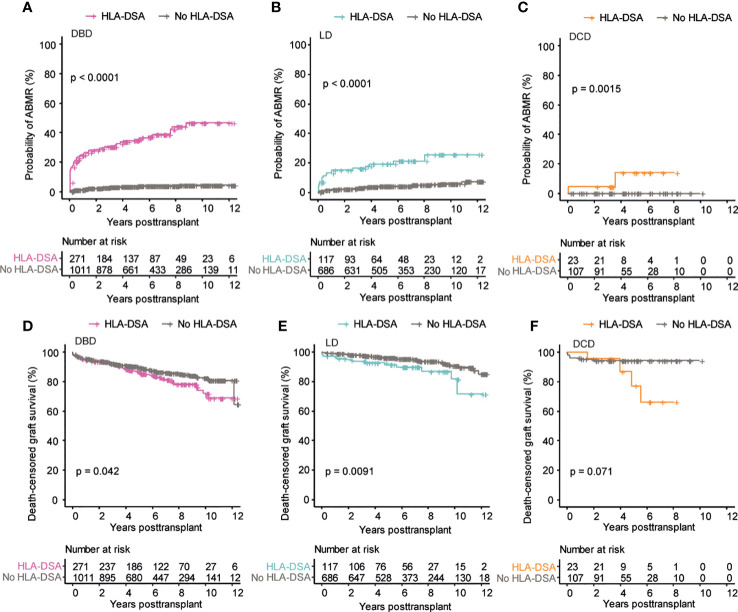
Pre-transplant DSA significantly impacts transplantation outcome in all the three donation types. Cumulative incidence of ABMR in patients who received DBD transplants **(A)**, LD transplants **(B)**, and DCD transplants **(C)**. Cumulative incidence of death-censored graft survival in patients who received DBD transplants **(D)**, LD transplants **(E)**, and DCD transplants **(F)**. Log-rank test was used to test p value of the Kaplan-Meier survival curves for all the graphs.

We next investigated if the presence of pre-transplant DSA differentially affected the outcome of kidney transplantation concerning the type of donation. The probability of developing ABMR was highest among DSA positive recipients receiving a DBD kidney transplant (**Figure 3A**), but DSA positive recipients of LD and DCD transplants also showed a significant increase in ABMR as compared to recipients without DSA (**Figures 3B, C**). The presence of pre-transplant DSA was, however, not associated with an increased risk of TCMR (**Supplementary Figures 2A–C**). For graft loss, the strongest impact of pre-transplant DSA was seen in the recipients of an LD graft, likely due to the excellent graft survival in DSA negative recipients within this group (**Figure 3E**). Recipients of DBD grafts with DSA also showed a significantly decreased graft survival whereas there was a strong trend towards increased graft loss in DSA positive DCD recipients without reaching statistical significance, likely due to the smaller number of patients within this group (**Figures 3D, F** and **Table 2**). The presence of DSA was also associated with accelerated decline in graft function as measured by eGFR slope in DBD transplants (**Supplementary Figures 2D–I**). In summary, the presence of pre-transplant DSA was associated with increased risk for ABMR and graft loss in all the investigated donation types.

**Table 2:** Summary of the investigated outcomes after kidney transplantation with and withour pre-transplant DSA, and the induction therapy applied on the DSA patients.

Parameter	DBD total patients (n=1282)	DSA (n=271)	No DSA (n=1011)	p value (DSA vs. No DSA)
ABMR	9.3%	30.6%	3.6%	<0.0001
TCMR	18.6%	15.9%	19.4%	0.006
Graft loss	13.8%	17.7%	12.8%	<0.0001
Mean eGFR (ml/min/1.73m2)	50.76	49.82	51.69	0.967
Mean eGFR slope (ml/min/1.73m2/year)	-0.84	-1.27	-0.42	0.013
Mean total eGFR slope (ml/min/1.73m2/year)	-1.25	-1.65	-0.85	0.024
**Induction therapy**				
ATG/Thymo	24.5%	64.9%	13.6%	<0.0001
Basiliximab	73.6%	34.7%	84.1%	<0.0001
No induction	1.9%	0.4%	2.3%	<0.0001
Parameter	LD total patients (n=803)	DSA (n=117)	No DSA (n=686)	p value (DSA vs. No DSA)
ABMR	6.6%	11.9%	4.7%	<0.0001
TCMR	16.1%	18.8%	15.6%	0.092
Graft loss	6.7%	11.1%	6.0%	<0.0001
Mean eGFR (ml/min/1.73m2)	52.53	50.10	54.95	0.638
Mean eGFR slope (ml/min/1.73m2/year)	-0.36	-0.28	-0.44	0.793
Mean total eGFR slope (ml/min/1.73m2/year)	-0.71	-0.72	-0.70	0.682
**Induction therapy**				
ATG/Thymo	17.6%	67.5%	9.0%	<0.0001
Basiliximab	78.3%	32.5%	86.2%	<0.0001
No induction	4.1%	0.0%	4.8%	<0.0001
Parameter	DCD total patients (n=130)	DSA (n=23)	No DSA (n=107)	p value (DSA vs. No DSA)
ABMR	1.5%	8.7%	0.0%	<0.0001
TCMR	8.5%	4.3%	9.3%	0.106
Graft loss	7.7%	17.4%	5.6%	<0.0001
Mean eGFR (ml/min/1.73m2)	54.67	58.86	50.49	0.820
Mean eGFR slope (ml/min/1.73m2/year)	-0.32	-0.85	0.21	0.266
Mean total eGFR slope (ml/min/1.73m2/year)	-0.86	-1.61	-0.12	0.408
**Induction therapy**				
ATG/Thymo	72.3%	87.0%	69.2%	<0.0001
Basiliximab	26.9%	13.0%	29.9%	<0.0001
No induction	0.8%	0.0%	0.9%	0.352

### Similar outcomes in DSA positive patients between donation types

**Figure 4:**
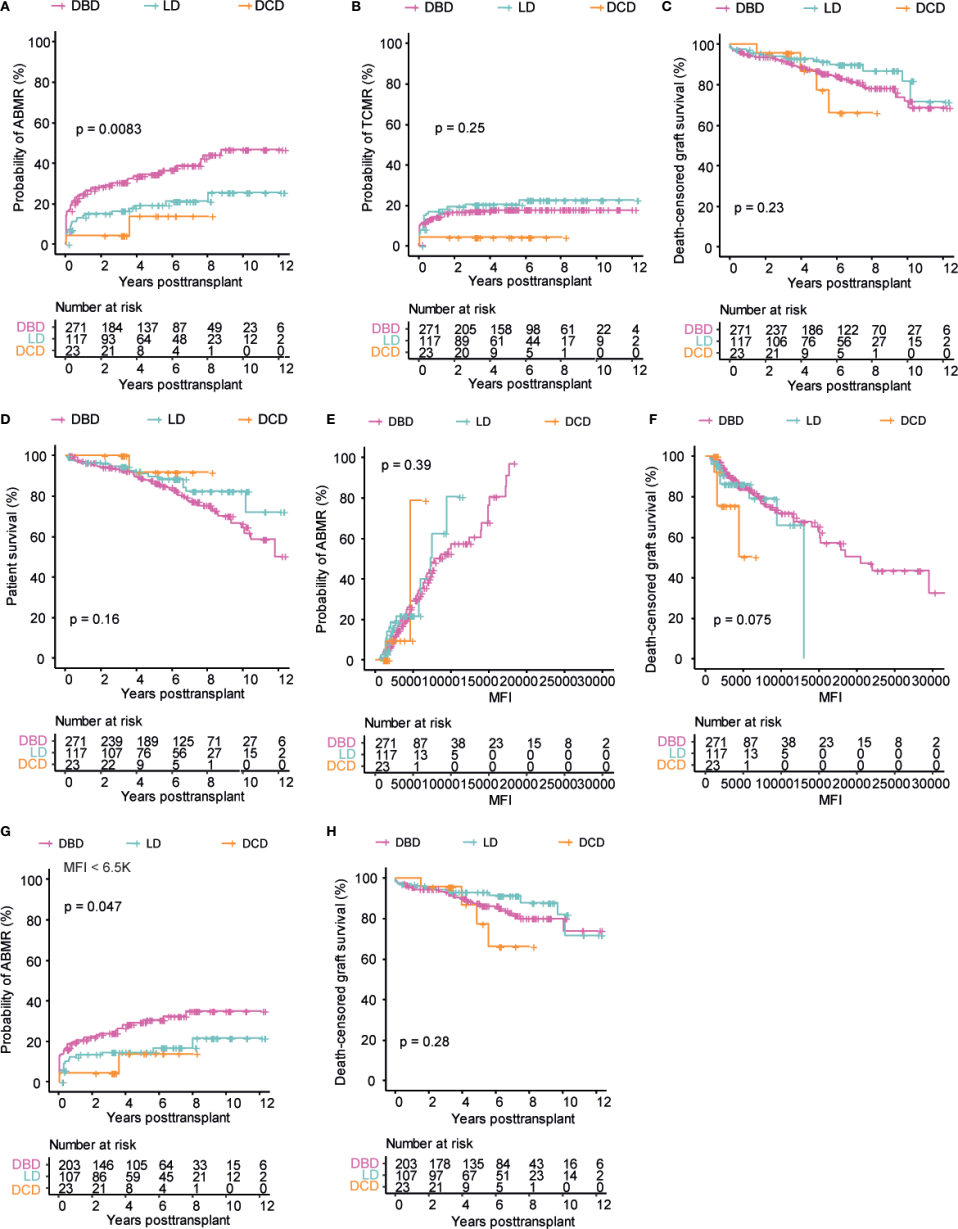
Comparable outcome in DSA positive patients among three different donation types. Cumulative incidence of ABMR **(A)**, TCMR **(B)**, patient survival **(C)**, and death-censored graft survival **(D)** over time in DSA positive patients. Cumulative incidence of ABMR **(E)**, and death-censored graft survival **(F)** with regards to cumulative DSA MFI value. Cumulative incidence of ABMR **(G)**, and death-censored graft survival **(H)** in patients with a DSA MFI value below 6.5k. Log-rank test was used to test p value of the Kaplan-Meier survival curves for all the graphs.

In order to directly compare the effect of DSA in the different donation types, we next focused on the subgroup of DSA positive patients. The risk of developing AMBR was significantly higher in DSA positive DBD recipients as compared to both LD and DCD recipients (**Figure 4A**). The same trend was, however, not seen in the setting of TCMR where both DBD and LD recipients appeared to have very similar risk whereas DSA positive DCD recipients appeared to have a somewhat lower risk (**Figure 4B**). In terms of graft loss, all of the DSA positive patients regardless of donation type showed very similar death censored graft survival (**Figure 4C**), with a slight trend towards inferior graft survival in DSA positive DCD recipients beyond 5 years, but this was based on very few patients and did not reach significance. In DSA negative patients, there was a significantly increased risk of TCMR and graft loss associated with DBD transplants whereas the risk of ABMR as well as eGFR decline was relatively similar between the donation types (**Supplementary Figures 3A–E**). The overall patient survival in DSA positive patients was not significantly different between the different donation types, which is in contrast to the differences observed for the whole population (**Figures 2F** and **4D**). The risk of both ABMR and graft loss was strongly associated with the cumulative MFI of the DSA in all donation types (**Figures 4E, F**). In order to investigate if the differences in the cumulative DSA MFI between the different types of donation (**Figure 1B**) impacted our comparison of ABMR and graft loss in the different DSA positive patients, we performed a subgroup analysis of patients with a cumulative DSA MFI of <6500. This allowed us to include all DSA positive DCD transplants and compare them to DBD and LD transplants with cumulative DSA in the same range. This additional analysis resulted in a more similar risk for ABMR being observed between the different donation types, even though DBD recipients still showed a significantly higher risk (**Figure 4G**). Concerning graft survival, the exclusion of high cumulative MFI transplants in the DBD and LD positive group did not markedly affect the comparison and there were no marked differences in eGFR slope (**Figure 4H** and **Supplementary Figures 5E, F**). A further analysis including patients with a cumulative DSA MFI of <1000 showed a trend for better outcome in DCD transplants (**Supplementary Figures 5A–D**). In summary, outcome was similar for DSA positive transplants regardless of donation type and a higher cumulative DSA MFI was strongly associated with increased risk for ABMR and graft loss.

### The presence of Class II directed pre-transplant DSA are consistently associated with worse outcome regardless of the type of donation

**Figure 5:**
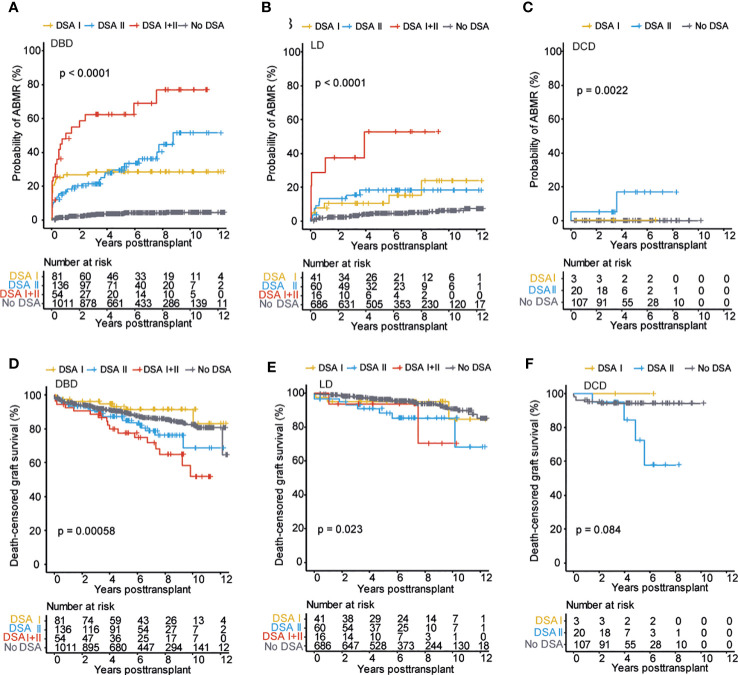
Class II directed pre-transplant DSA is associated with worse outcomes regardless of donation type. Cumulative incidence of ABMR in patients who received DBD transplants **(A)**, LD transplants **(B)**, and DCD transplants **(C)** stratified on DSA target HLA Class. Cumulative incidence of death-censored graft survival in patients who received DBD transplants **(D)**, LD transplants **(E)**, and DCD transplants **(F)** stratified on DSA target HLA Class. Log-rank test was used to test p value of the Kaplan-Meier survival curves for all the graphs.

In order to examine if the target antigen HLA Class of the detected DSA differentially impacted transplant outcome based on the type of donation we investigated this within our cohort. In DBD transplants, DSA directed against Class I and Class II showed a similarly increased risk of ABMR, whereas recipients with a combination of DSA against both Class I + Class II showed the highest risk (**Figure 5A**). This was also the case in the setting of LD transplants (**Figure 5B**) however in the setting of DCD transplants we could only detect an increased risk of ABMR associated with Class II DSA (**Figure 5C**). This was likely in part related to the limited number of patients that revived a DCD transplant in the setting of a pre-transplant Class I DSA (n=3). Concerning graft loss, we could only detect an increased risk of graft loss in the presence of Class II DSA or in the setting of combined Class I and Class II DSA across all donation types (**Figures 5D–F**). DSA against Class I or Class II were not associated with increased risk of TCMR in any of the donation types (**Supplementary Figures 4A–C**). Recipients with DSA against Class II or a combination of Class I and Class II DSA showed evidence of accelerated decline in graft function as measured by eGFR slope across donation types (**Supplementary Figures 4D–I**). In summary, Class II directed DSA were associated with a similar worse transplant outcome across all donation types.

### Prolonged cold ischemia time is associated with worse outcome in DSA positive transplants across donation types

**Figure 6:**
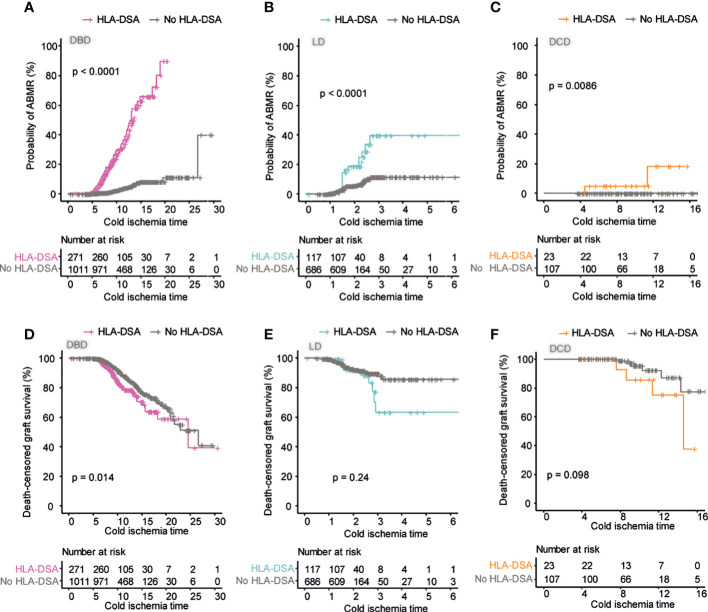
Prolonged cold ischemia time has a large influence on ABMR risk in DSA positive patients. Cumulative incidence of ABMR in patients who received DBD transplants **(A)**, LD transplants **(B)**, and DCD transplants **(C)** in relation to cold ischemia time. Cumulative incidence of death-censored graft survival in patients who received DBD transplants **(D)**, LD transplants **(E)**, and DCD transplants **(F)** in relation to cold ischemia time. Log-rank test was used to test p value of the Kaplan-Meier survival curves for all the graphs.

Since we could not find strong evidence for an additive effect of pre-transplant DSA and extended warm ischemia time in the setting of DCD transplantation within our cohort, we next wanted to investigate if prolonged cold ischemia time could be an additive risk factor in the setting of pre-transplant DSA. We analyzed the risk of ABMR in DSA positive and negative transplants within the different donation types according to cold ischemia time. In DBD recipients, we could observe an effect of extended cold ischemia time on the risk of AMBR in both DSA negative and positive patients (**Figure 6A**). This effect appeared to be magnified in DSA positive patients where the combination of pre-transplant DSA and extended cold ischemia time posed a very large risk for ABMR. This effect was also visible in the setting of LD transplants even though the variability in cold ischemia time was, as expected, markedly smaller (**Figure 6B**). For DCD transplants, there was interestingly no episode of ABMR detected in DSA negative patients regardless of cold ischemia time (**Figure 6C**). In DSA positive DCD transplants prolonged cold ischemia time appeared to have a significant effect on the risk of ABMR (**Figure 6C**). In order to investigate if extended cold ischemia time was also associated with accelerated graft loss in DSA positive transplants, we investigated this stratified on donation type. For DBD donors, the extended cold ischemia time was correlated to increased risk for graft loss in both DSA negative and positive transplants but the risk was significantly increased in patients with DSA as compared to DBD transplants without DSA (**Figure 6D**). A similar trend could be observed for both LD and DCD transplants but this did not reach statistical significance possibly due to the low number of DSA positive transplants with extended cold ischemia time (**Figures 6E, F**). In summary, extended cold ischemia time was associated with an increased risk for ABMR and graft loss in DSA positive as compared to DSA negative transplants.

### Multivariate analysis of risk factors for graft loss show similar pattern in DBD, LD and DCD transplants

**Figure 7:**
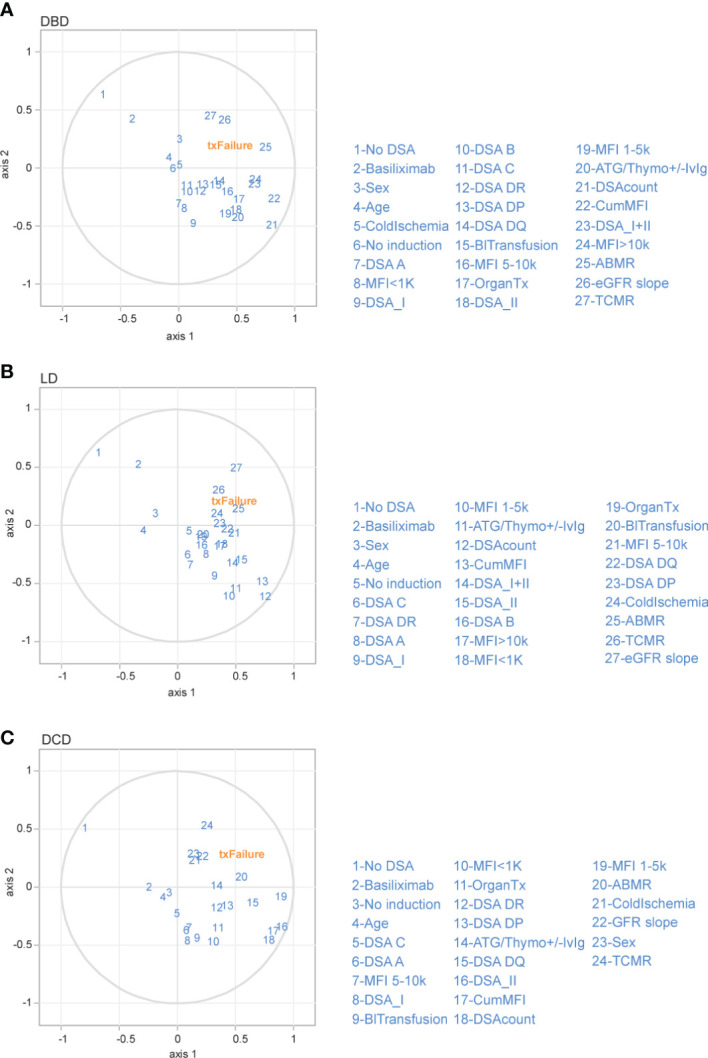
Partial least squares (PLS) regression exploratory biplot for the first two components describing the correlation among different immunological risk factors to graft loss in the different donation types. Correlation shown between graft loss (txFailure) and the risk factors (in blue numbers) for DBD **(A)**, LD **(B)** and DCD **(C)** transplants. The first two axes which correspond to PLS components 1 and 2 are shown. The distance between the individual risk factors and the center indicates the strength of the correlation with each component and their alignments represent the correlation they contribute to the variation explained by each component. (OrganTx, Organ Transplantation; BlTransfusion, Blood Transfusion; DSAcount, Number of DSA).

In order to perform multivariate analyses on factors associated with graft loss in an exploratory fashion and circumvent the high degree of collinearity between several of the investigated DSA variables we performed an exploratory partial least squares regression (PLS) model. With the PLS model, we investigated factors that could be associated with graft loss in an exploratory fashion that we previously investigated in a univariate analysis within the three different donation type groups. As expected eGFR decline, ABMR and TCMR fell in the same quadrant as graft loss (txFailure), indicating that they were more closely aligned together and influenced by the similar risk factors explained by both component 1 and 2 in all of the donation types (**Figures 7A–C**). The presence of DSA against Class II antigens as well as multiple DSA and MFI were also associated with graft loss (**Figures 7A–C**). DSA against Class II (particularly HLA-DQ) were also strongly coupled to AMBR in an additional exploratory PLS analysis in all donation type groups (**Supplementary Figures 6A–C**). A univariate cox regression analysis for factors associated with ABMR (**Table 3**) and graft loss (**Table 4**) was also performed. In summary, our multivariate exploratory analysis shows a similar pattern of risk factors associated with graft loss and ABMR across all donation types.

**Table 3:** Univariate analysis to identify potential predictors of ABMR.

DBD			
	Univariate analysis		
Parameter	HR	95% CI	p value
Recipient age	0.981	0.967 - 0.994	0.004
Recipient gender	0.705	0.492 - 1.012	0.058
Donor age	0.998	0.988 - 1.007	0.591
Donor gender	0.798	0.557-1.143	0.218
Cold ischemia time	0.961	0.917 - 1.006	0.091
DSA	10.160	6.865 - 15.020	**<0.001**
DSA class			
No DSA	Reference		
DSA I	7.909	4.578 - 13.660	**<0.001**
DSA II	8.756	5.531 - 13.860	**<0.001**
DSA I+II	18.400	11.100 - 30.500	**<0.001**
Induction therapy			
ATG/Thymo	2.706	1.886-3.883	**<0.001**
Basiliximab	0.412	0.287-0.591	**<0.001**
LD			
	Univariate analysis		
Parameter	HR	95% CI	p value
Recipient age	0.991	0.973 - 1.010	0.354
Recipient gender	0.518	0.302 - 0.887	**0.017**
Donor age	1.011	0.986 - 1.037	0.388
Donor gender	1.465	0.853 - 2.517	0.166
Cold ischemia time	1.115	1.061 - 1.172	**<0.001**
DSA	4.588	2.642 - 7.966	**<0.001**
DSA class			
No DSA	Reference		
DSA I	3.435	1.436 - 8.217	0.006
DSA II	3.884	1.851 -8.149	**<0.001**
DSA I+II	11.9	4.952 -28.597	**<0.001**
Induction therapy			
ATG/Thymo	2.115	1.176-3.802	**0.012**
Basiliximab	0.577	0.324-1.027	0.062
DCD			
	Univariate analysis		
Parameter	HR	95% CI	p value
Recipient age	1.161	0.935 - 1.442	0.177
Recipient gender	0.773	0.048 - 12.42	0.856
Donor age	0.953	0.883 - 1.029	0.217
Donor gender	0.696	0.043 - 11.170	0.799
Cold ischemia time	0.910	0.577- 1.437	0.687
DSA	No ABMR was developed when DSA was abscent		
DSA class			
No DSA	/		
DSA I	/		
DSA II	/		
Induction therapy	/		
ATG/Thymo	0.374	0.023-5.982	0.487
Basiliximab	2.827	0.177-45.22	0.463

HR, Hazard ratio; CI, confidence interval. Bold values indicate significance.

**Table 4:** Univariate analysis to identify potential predictors of graft loss.

DBD			
	Univariate analysis		
Parameter	HR	95% CI	p value
Recipient age	1.006	0.994-1.018	0.302
Recipient gender	1.256	0.916-1-721	0.157
Donor age	1.021	1.012-1.030	**<0.001**
Donor gender	0.722	0.538-0.970	0.031
Cold ischemia time	0.991	0.957-1-026	0.591
DSA	1.408	1.011-1.961	0.043
DSA class			
No DSA	Reference		
DSA I	0.622	0.291-1.331	0.221
DSA II	1.517	0.981-2.348	0.061
DSA I+II	2.427	1.463-4.025	**<0.001**
Induction therapy			
ATG/Thymo	1.328	0.964-1.829	0.083
Basiliximab	0.797	0.581-1.095	0.161
Rejections			
ABMR	3.311	2.353-4.659	**<0.001**
TCMR	2.019	1.473-2.768	**<0.001**
DCD			
	Univariate analysis		
Parameter	HR	95% CI	p value
Recipient age	0.98	0.937-1.024	0.367
Recipient gender	3.205	0.677-15.170	0.142
Donor age	1.03	0.979-1.084	0.26
Donor gender	1.644	0.424-6.371	0.472
Cold ischemia time	1.136	0.953-1.355	0.154
DSA	3.027	0.854-10.730	0.086
DSA class			
No DSA	/		
DSA I	/		
DSA II	/		
DSA I+II	/		
Induction therapy			
ATG/Thymo	3.272	0.414-25.880	0.261
Basiliximab	0.317	0.040-2.509	0.277
Rejections			
ABMR	6.679	0.841-53.050	0.073
TCMR	5.963	1.528-23.270	0.01
Living related			
	Univariate analysis		
Parameter	HR	95% CI	p value
Recipient age	1.002	0.983-1.021	0.835
Recipient gender	0.765	0.444-1.317	0.334
Donor age	1.026	1.000-1.052	0.054
Donor gender	1.323	0.772-2.270	0.309
Cold ischemia time	1.045	1.006-1.085	0.023
DSA	2.251	1.204-4.209	0.011
DSA class			
No DSA	Reference		
DSA I	1.325	0.410-4.283	0.638
DSA II	2.851	1.335-6.097	0.007
DSA I+II	2.854	0.687-11.855	0.149
Induction therapy			
ATG/Thymo	1.332	0.701-2.530	0.381
Basiliximab	0.935	0.500-1.747	0.832
Rejections			
ABMR	3.174	1.635-6.161	**<0.001**
TCMR	2.427	1.385-4.256	0.002

HR, Hazard ratio; CI, confidence interval. Bold values indicate significance.

## Discussion

Previous large studies on the impact of pre-transplant DSA on transplant outcome stratified by donation type have shown somewhat conflicting results relating to the impact on LD and deceased donor transplant outcome (5, 10). Both of these studies also showed a similar risk associated with DSA directed at Class I and Class II HLA antigens. Both of these studies were however not based on a complete vXM and the study from Ziemann et al. also had a somewhat shorter follow-up time. Our study, which is the first with a complete vXM and an extended follow-up time, show comparable risk of pre-transplant DSA across all donation type. Furthermore, we find significantly increased risk for ABMR associated with both Class I and Class II DSA across donation type, whereas only DSA directed at Class II antigens was associated with a significantly increased long-term graft loss as well as with increased decline in graft function. We were also in contrast to the study by Ziemann et al. able to show a large effect of DSA MFI both related to AMBR and graft loss across donation types. We were also in our cohort able, for the first time, to evaluate the impact of pre-transplant DSA in the setting of DCD transplantation. Many transplant centers are reluctant to perform transplantations in DSA positive patients with DCD donors due to a perceived additive risk of the extended warm ischemia time and pre-existing alloimmunity. With the increased amount of deceased donor transplantations using DCD donors, this further limits the possibilities for alloimmunized patients. Our investigation of 130 DCD kidney transplantation (23 DSA positive) with a complete vXM within the STCS did not show a significant increased risk of ABMR or graft loss as compared to DSA positive DBD transplants (271 DSA positive). On the contrary, we found evidence of decreased amounts of ABMR and superior graft function in DSA positive DCD transplants as compared to DSA positive DBD transplants. The trend for superior outcomes in terms of ABMR, TCMR and graft function seen in DCD transplants could have several explanations. A large majority of DCD transplants received induction therapy with ATG (72%) even though 82% were DSA negative. Furthermore, the DCD donors especially early at the start of the DCD program were likely to have had a significantly lower kidney donor profile index even though this data is not available within the STCS (20). The combination of better donors and more intensive induction therapy might have led to the improved outcomes observed and the negative effect of DSA reported in our study should be evaluated in this context.

We could not find any evidence of an additive negative effect associated with the increased warm ischemia time in the setting of DCD transplantation and pre-transplant DSA. We were, however, able to find an increased risk of DSA associated with extended cold ischemia time. This was visible both when analyzing the risk of ABMR as well as graft loss where extended cold ischemia was associated with significantly worse graft survival in DBD transplants. Previous studies on the impact of Class II DSA in relation to cold ischemia time in mice have also shown worse outcome in the setting of longer cold ischemia time (21). Previous human studies have also shown that DSA positive patients with delayed graft function (DGF) have a significantly worse graft survival and a higher incidence of ABMR, and that highly immunized patients particularly benefit from shortening of cold ischemia time (22, 23). It is also well established that longer cold ischemia time is associated with higher incidence of DGF and as such, our findings build on previous literature and suggest that cold ischemia time should be considered when performing DSA positive transplants (24–26). However, the impact of DGF on graft survival remains somewhat unclear as previous literature has shown a two-fold higher incidence of DGF in DCD transplants, but transplant outcome is comparable between DCD and DBD transplants in many centers (14, 27, 28). The high incidence of ABMR seen in patients with DSA and extended cold ischemia time might also in part be influenced by the increased amounts of biopsies that are performed in the settings of DGF and the importance of the presence of DSA for the classification of rejection according to the Banff criteria.

Our study has several strengths including the complete vXM, the long follow-up time and the excellent quality of the data on outcome and biopsy proven rejection as well as kidney function. Our study also has several limitations related to the multicenter design and long period of inclusion of patients, including differences in maintenance and induction immunosuppressive therapies at different centers, as well as associated with the evaluation of SAB results and individual routines for the diagnosis and therapy of rejection. The relatively small number of DSA positive DCD transplants as well as the somewhat shorter follow-up time in this group makes it more difficult to interpret the result. Additionally we cannot exclude the possibility that there are differences in the donor quality between DCD and DBD donors that might also affect our results and our ability to identify clinically relevant risks. Data on the development of *de novo* DSA or anti-HLA antibody kinetics post transplantation is unfortunately not captured in the STCS database and we are unable to evaluate the effect of these essential markers on the outcome of transplantation.

In summary, we have performed the largest study to date with a complete vXM, on the impact of pre-transplant DSA on the outcome of kidney transplantations stratified by donation type. Our data show similar effects of DSA across all donation types and argue that the immunological risk associated with pre-transplant DSA could be performed in a similar way regardless of donation type. This suggests that the same MFI cut-offs and DSA risk scores currently used in the DBD setting could also be utilized in the setting of DCD donation. We also found evidence for an additive negative impact of extended cold ischemia time in the setting of pre-transplant DSA positive patients, which suggest that cold ischemia time might be an important factor to consider when performing DSA positive transplants.

## The members of the Swiss Transplant Cohort Study 

Patrizia Amico, Andres Axel, John David Aubert, Vanessa Banz, Beckmann Sonja, Guido Beldi, Christoph Berger, Ekaterine Berishvili, Isabelle Binet, Pierre-Yves Bochud, Sanda Branca, Heiner Bucher, Thierry Carrel, Emmanuelle Catana, Yves Chalandon, Sabina De Geest, Olivier De Rougemont, Michael Dickenmann, Joëlle Lynn Dreifuss, Michel Duchosal, Thomas Fehr, Sylvie Ferrari-Lacraz, Nicola Franscini, Christian Garzoni, Paola Gasche Soccal, Christophe Gaudet, Déla Golshayan, Nicolas Goossens, Karine Hadaya, Jörg Halter, Dominik Heim, Christoph Hess, Sven Hillinger, Hans Hirsch, Patricia Hirt, Günther Hofbauer, Uyen Huynh-Do, Franz Immer, Michael Koller (Head of the data center), Mirjam Laager, Bettina Laesser, Roger Lehmann, Alexander Leichtle, Christian Lovis, Oriol Manuel, Hans-Peter Marti, Pierre Yves Martin, Michele Martinelli, Valérie McLin, Katell Mellac, Aurelia Mercay, Karin Mettler, Nicolas Mueller (Chairman Scientific Committee), Antonia Müller, Thomas Müller, Ulrike Müller-Arndt, Beat Müllhaupt, Mirjam Nägeli, Graziano Oldani, Manuel Pascual (Executive office), Klara Posfay-Barbe, Juliane Rick, Anne Rosselet, Simona Rossi, Silvia Rothlin, Frank Ruschitzka, Urs Schanz, Stefan Schaub, Aurelia Schnyder, Macé Schuurmans, Thierry Sengstag, Federico Simonetta, Katharina Staufer, Susanne Stampf, Jürg Steiger (Head, Executive office), Guido Stirniman, Ueli Stürzinger, Christian Van Delden (Executive office), Jean-Pierre Venetz, Jean Villard, Julien Vionnet,Madeleine Wick (STCS coordinator), Markus Wilhlem, Patrick Yerly.

## Data availability statement

The raw data supporting the conclusions of this article will be made available by the authors, without undue reservation.

## Ethics statement

The studies involving human participants were reviewed and approved by The Cantonal Ethics Committee of Zurich (BASEC-Nr.2021-0083). The patients/participants provided their written informed consent to participate in this study.

## Author contributions

OD and YD collected and analyzed data and wrote the manuscript. CW, LF, JV, SF-L, DG, MG, IB, UW, DS, TS, and SS collected data and critically reviewed the manuscript. JN designed the research, collected and analyzed the data, and wrote the manuscript. All authors contributed to the article and approved the submitted version.

## Supplementary material

Supplementary Figure 1 Transplant outcome in DBD and DCD patients who received ATG as the induction therapy but not Cyclosporine A as the maintenance immunosuppressant medication. Cumulative incidence of ABMR **(A)**, TCMR **(B)**, and death-censored graft survival **(C)** in the DBD and DCD patients. Log-rank test was used to test p value of the Kaplan-Meier survival curves for **(A–C)**.

Supplementary Figure 2 Comparable TCMR risk in patients with pre-transplant DSA but DSA positive DBD recipients show accelerated decline in graft function. Cumulative incidence of TCMR in patients who received DBD transplants **(A)**, LD transplants **(B)**, and DCD transplants **(C)**. The total mean slope of eGFR **(D, F, H),** and the collective longitudinal mean annual slope of eGFR **(E, G, I)**. Log-rank test was used to test p value of the Kaplan-Meier survival curves for **(A-C)**. A Mann-Whitney U test was used for **(D, F, H)**, and one-way ANOVA analysis with Dunn’s *post hoc* test was used for **(E, G)**, and **(H)** to assess p values; **p<0.01, ***p<0.001.

Supplementary Figure 3 Transplant outocme in DSA negative patients and graft function in DSA positive patients. Cumulative incidence of ABMR **(A)**, TCMR **(B)**, and death-censored graft survival **(C)** in DSA negative patients for the different donation types. The total mean slope of eGFR **(D)**, and the collective longitudinal mean annual slope of eGFR **(E)** in DSA positive patients for the different donation types. Log-rank test was used to test p value of the Kaplan-Meier survival curves for **(A-C)**. One-way ANOVA analysis with Dunn’s *post hoc* test was used for **(D)** and two-way ANOVA analysis with Tukey’s multiple comparisons as a *post hoc* test was used for **(E)** to assess p values; *p<0.05.

Supplementary Figure 4 The impact of DSA directed against Class I and Class II on the outcome of kidney transplantation. Cumulative incidence of TCMR in patients who received DBD transplants **(A)** LD transplants **(B)**, and DCD transplants **(C)**. The total mean slope of eGFR **(D, F, H),** and the collective longitudinal mean annual slope of eGFR **(E, G, I)**. Log-rank test was used to test p value of the Kaplan-Meier survival curves for **(A-C)**. One-way ANOVA analysis with Dunn’s *post hoc* test was used for **(D, F, H)**, and two-way ANOVA analysis with Tukey’s multiple comparisons as *post hoc* test was used for **(E, G)**, and **(H)** to assess p values; *p<0.05, **p<0.01, ***p<0.001.

Supplementary Figure 5 Transplant outcome in DSA positive patients with a cumulative MFI value of <1K and <6.5K. Cumulative incidence of ABMR **(A)**, and death-censored graft survival **(B)** in patients with a cumulative MFI value <1K. The total mean slope of eGFR **(C, E),** and the collective longitudinal mean annual slope of eGFR **(D, F)**. Log-rank test was used to test p value of the Kaplan-Meier survival curves for **(A, B)**. One-way ANOVA analysis with Dunn’s *post hoc* test was used for **(C, E)**, and two-way ANOVA analysis with Tukey’s multiple comparisons as *post hoc* test was used for **(D)**, and **(F)** to assess p values; *p<0.05, **p<0.01, ***p<0.001.

Supplementary Figure 6 Partial least squares (PLS) regression biplot for the first two components describing the correlation among different immunological risk factors to the development of ABMR in the different donation types. Correlation shown between ABMR and the risk factors (in blue numbers) for DBD **(A)**, LD **(B)** and DCD **(C)** transplants. The first two axes which correspond to PLS components 1 and 2 are shown. The distance between the individual risk factors and the center indicates the strength of the correlation with each component and their alignments represent the correlation they contribute to the variation explained by each component. (OrganTx: Organ Transplantation, BlTransfusion: Blood Transfusion).
