# Supplementary figures and images for: Donation type and the effect of pre-transplant donor specific antibodies – Data from the Swiss Transplant Cohort Study
Source: Front Immunol. 2023 Feb 15;14:1104371. doi: 10.3389/fimmu.2023.1104371 (PMC9974644; doi:10.3389/fimmu.2023.1104371)

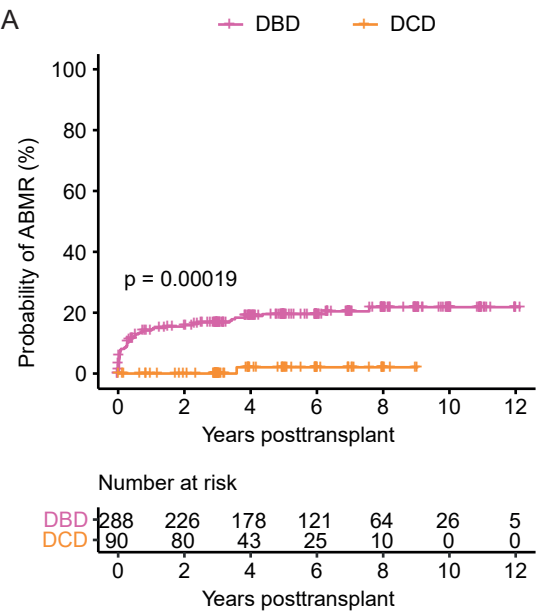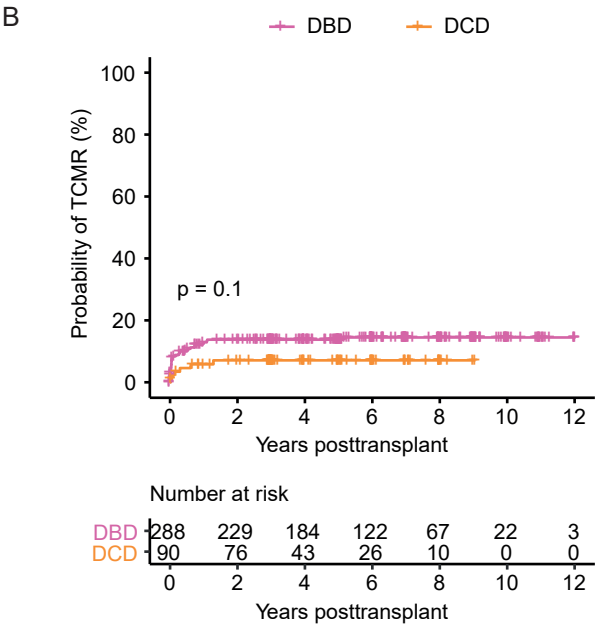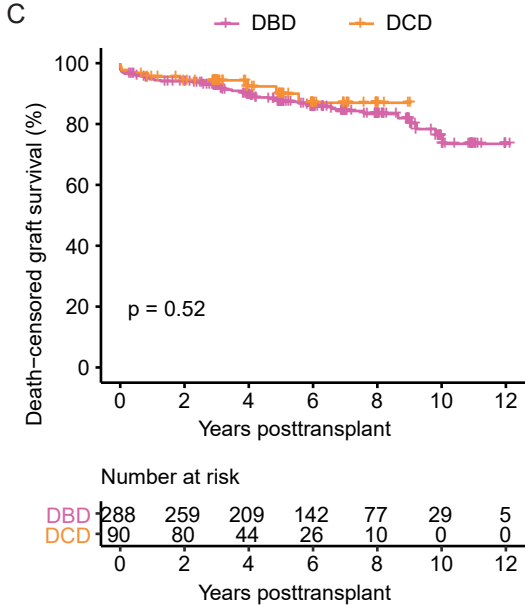

Supplement: Supplementary Figure 1 — Transplant outcome in DBD and DCD patients who received ATG as the induction therapy but not Cyclosporine A as the maintenance immunosuppressant medication. Cumulative incidence of ABMR (A), TCMR (B), and death-censored graft survival (C) in the DBD and DCD patients. Log-rank test was used to test p value of the Kaplan-Meier survival curves for (A–C). [file Image_1.pdf]

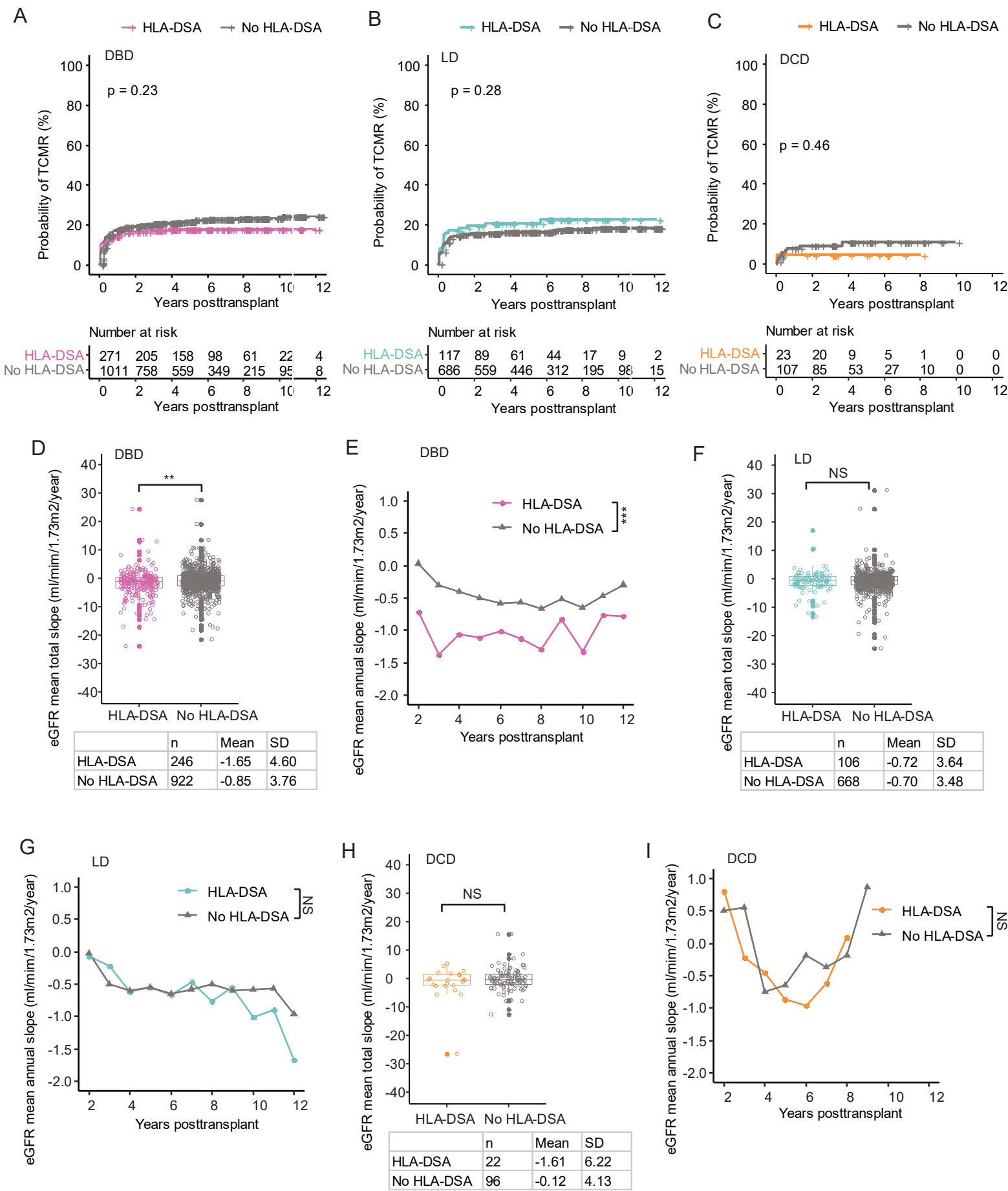

Supplement: Supplementary Figure 2 — Comparable TCMR risk in patients with pre-transplant DSA but DSA positive DBD recipients show accelerated decline in graft function. Cumulative incidence of TCMR in patients who received DBD transplants (A), LD transplants (B), and DCD transplants (C). The total mean slope of eGFR (D, F, H), and the collective longitudinal mean annual slope of eGFR (E, G, I). Log-rank test was used to test p value of the Kaplan-Meier survival curves for (A-C). A Mann-Whitney U test was used for (D, F, H), and one-way ANOVA analysis with Dunn’s post hoc test was used for (E, G), and (H) to assess p values; **p<0.01, ***p<0.001. [file Image_2.pdf]

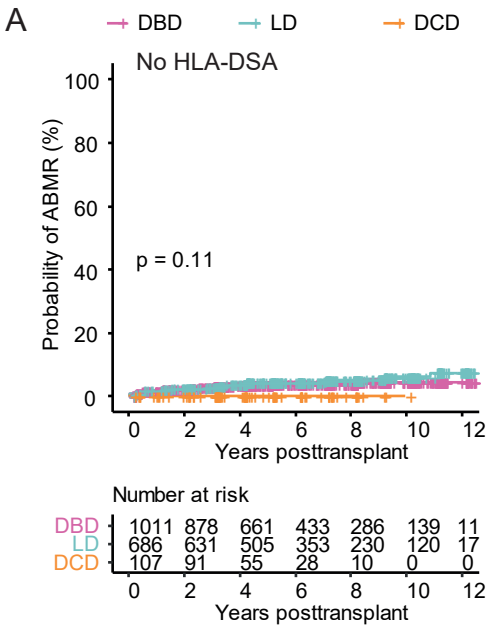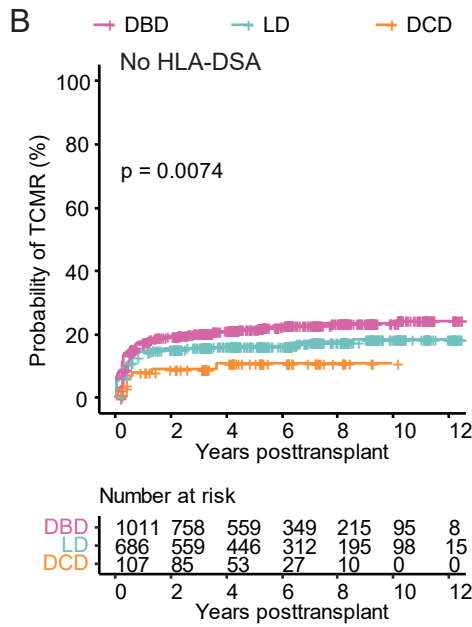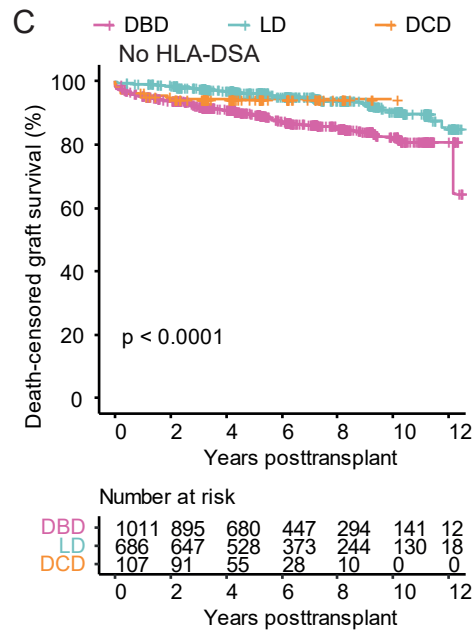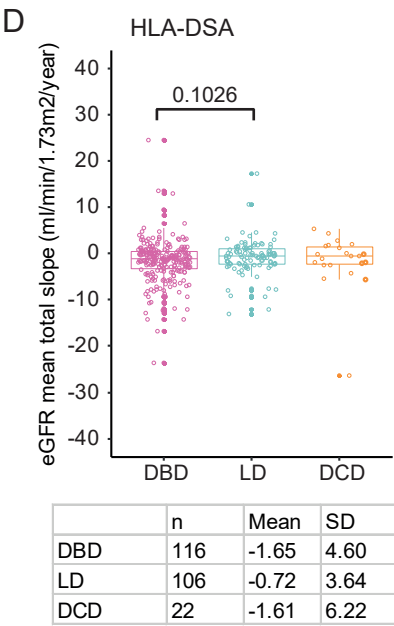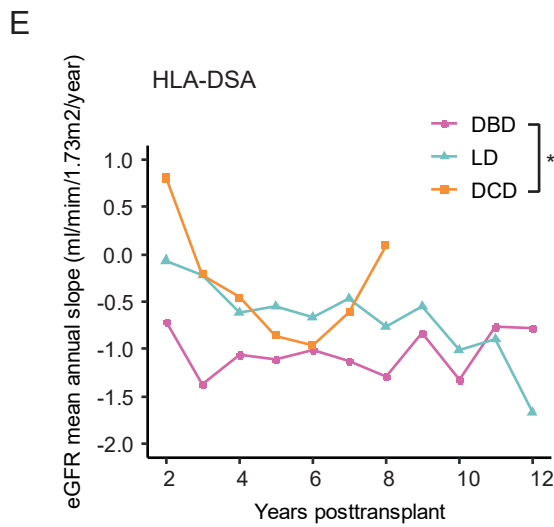

Supplement: Supplementary Figure 3 — Transplant outocme in DSA negative patients and graft function in DSA positive patients. Cumulative incidence of ABMR (A), TCMR (B), and death-censored graft survival (C) in DSA negative patients for the different donation types. The total mean slope of eGFR (D), and the collective longitudinal mean annual slope of eGFR (E) in DSA positive patients for the different donation types. Log-rank test was used to test p value of the Kaplan-Meier survival curves for (A-C). One-way ANOVA analysis with Dunn’s post hoc test was used for (D) and two-way ANOVA analysis with Tukey’s multiple comparisons as a post hoc test was used for (E) to assess p values; *p<0.05. [file Image_3.pdf]

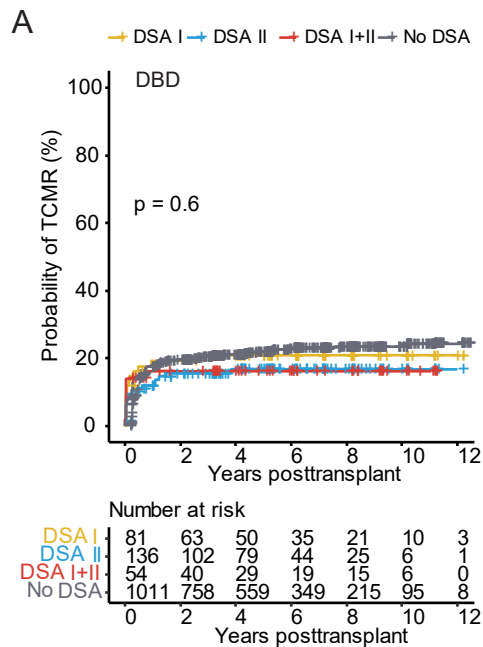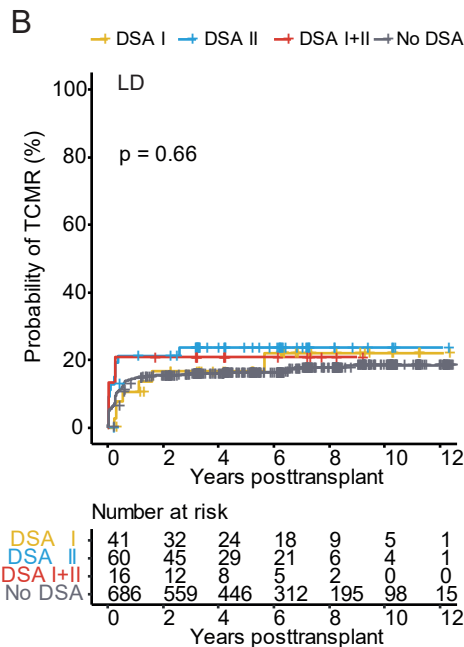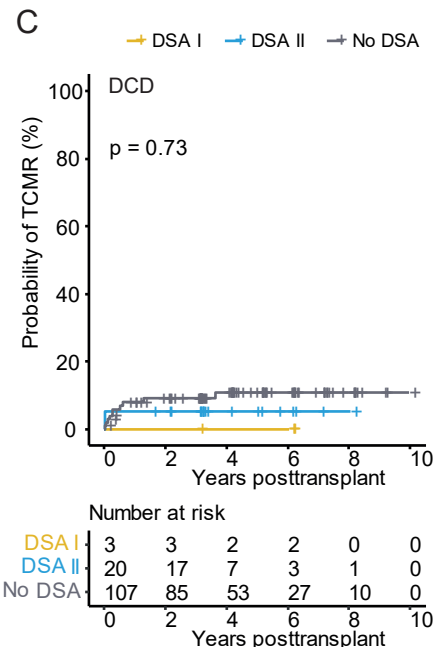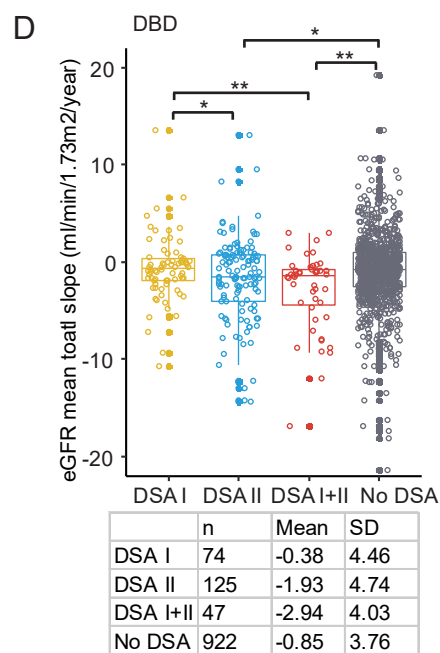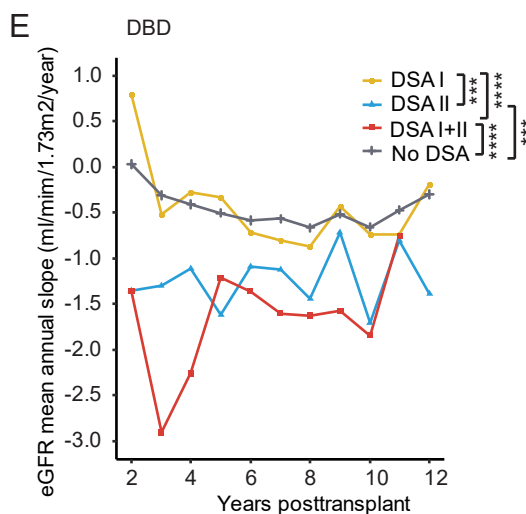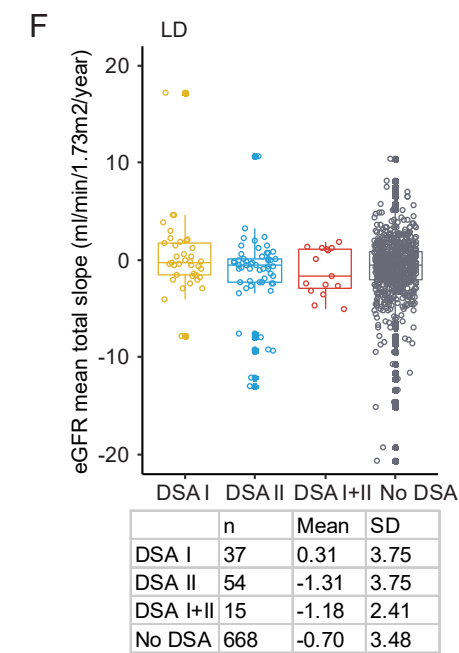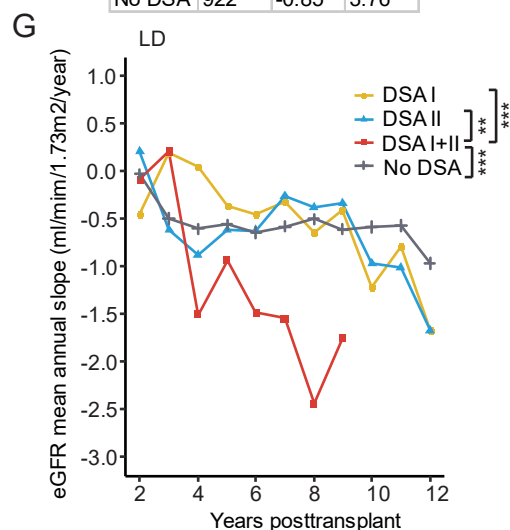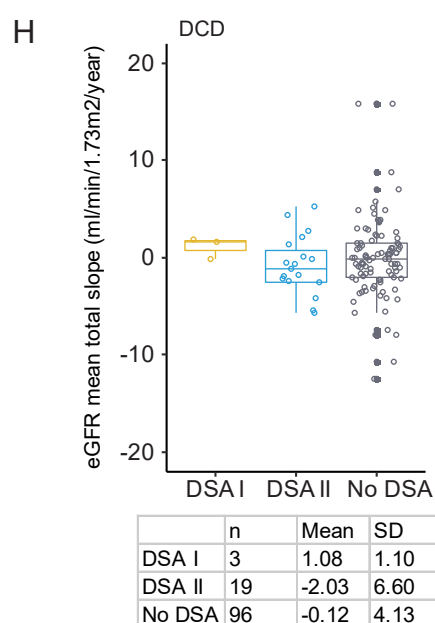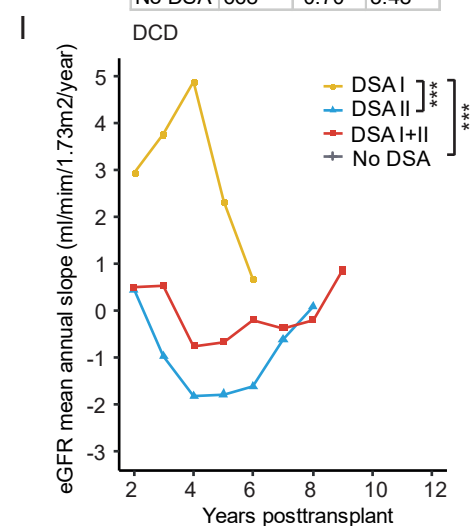

Supplement: Supplementary Figure 4 — The impact of DSA directed against Class I and Class II on the outcome of kidney transplantation. Cumulative incidence of TCMR in patients who received DBD transplants (A) LD transplants (B), and DCD transplants (C). The total mean slope of eGFR (D, F, H), and the collective longitudinal mean annual slope of eGFR (E, G, I). Log-rank test was used to test p value of the Kaplan-Meier survival curves for (A-C). One-way ANOVA analysis with Dunn’s post hoc test was used for (D, F, H), and two-way ANOVA analysis with Tukey’s multiple comparisons as post hoc test was used for (E, G), and (H) to assess p values; *p<0.05, **p<0.01, ***p<0.001. [file Image_4.pdf]

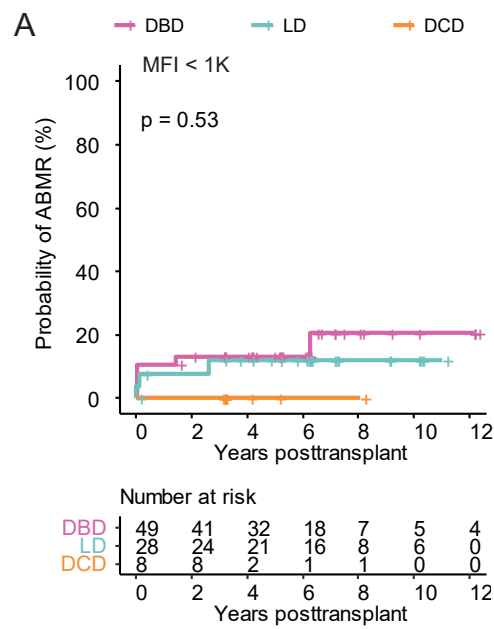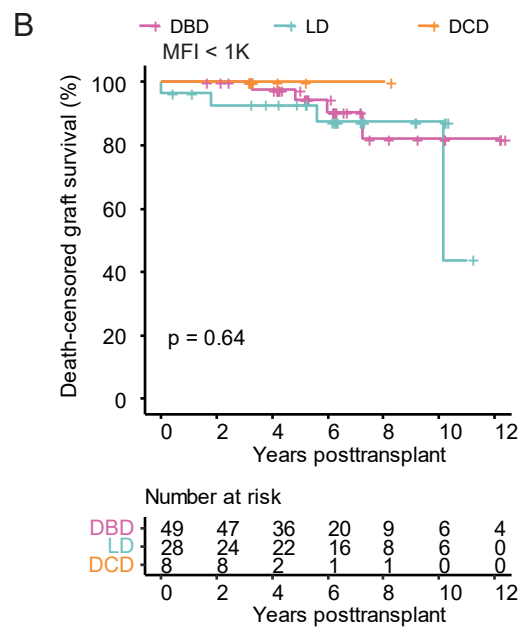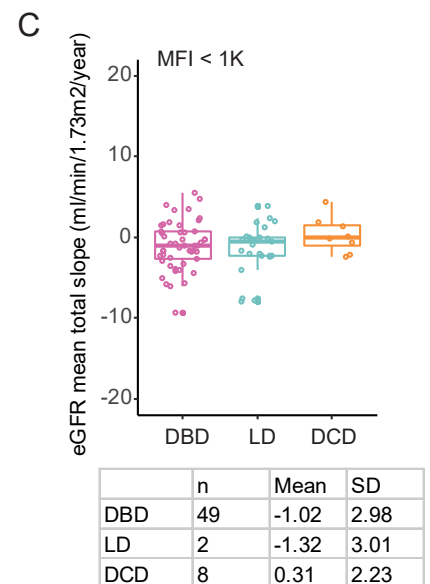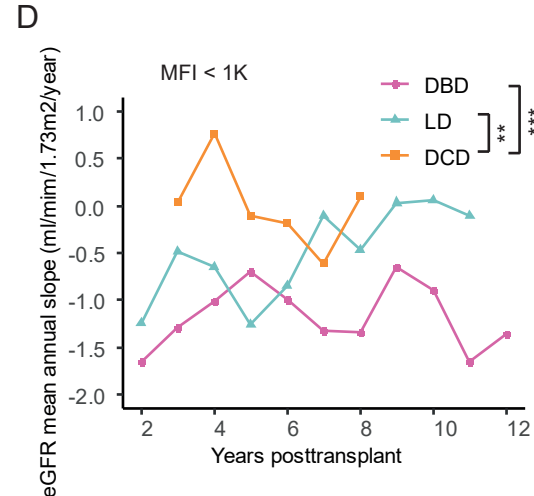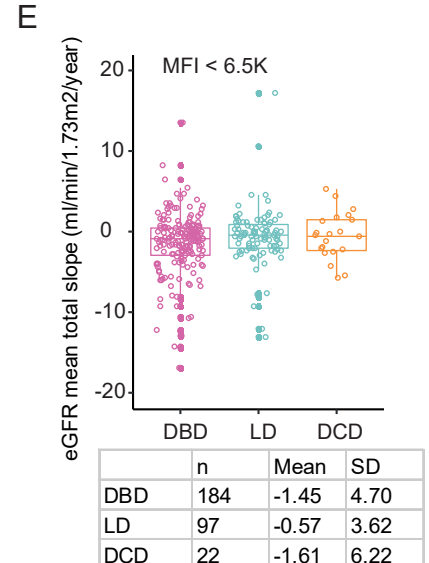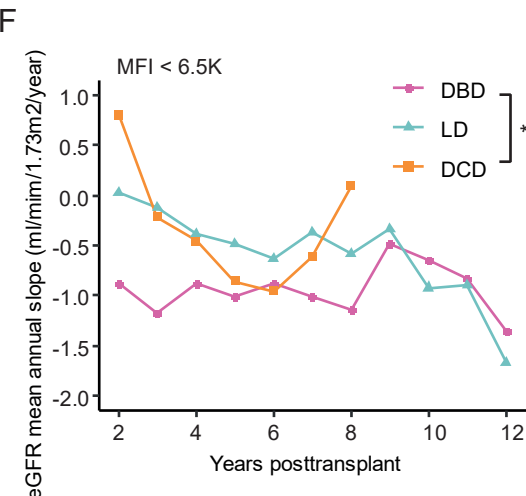

Supplement: Supplementary Figure 5 — Transplant outcome in DSA positive patients with a cumulative MFI value of <1K and <6.5K. Cumulative incidence of ABMR (A), and death-censored graft survival (B) in patients with a cumulative MFI value <1K. The total mean slope of eGFR (C, E), and the collective longitudinal mean annual slope of eGFR (D, F). Log-rank test was used to test p value of the Kaplan-Meier survival curves for (A, B). One-way ANOVA analysis with Dunn’s post hoc test was used for (C, E), and two-way ANOVA analysis with Tukey’s multiple comparisons as post hoc test was used for (D), and (F) to assess p values; *p<0.05, **p<0.01, ***p<0.001. [file Image_5.pdf]
