# Supplementary material for: Donation type and the effect of pre-transplant donor specific antibodies – Data from the Swiss Transplant Cohort Study
Source: Front Immunol. 2023 Feb 15;14:1104371. doi: 10.3389/fimmu.2023.1104371 (PMC9974644; doi:10.3389/fimmu.2023.1104371)
Supplement: Supplementary Figure 6 — Partial least squares (PLS) regression biplot for the first two components describing the correlation among different immunological risk factors to the development of ABMR in the different donation types. Correlation shown between ABMR and the risk factors (in blue numbers) for DBD (A), LD (B) and DCD (C) transplants. The first two axes which correspond to PLS components 1 and 2 are shown. The distance between the individual risk factors and the center indicates the strength of the correlation with each component and their alignments represent the correlation they contribute to the variation explained by each component. (OrganTx: Organ Transplantation, BlTransfusion: Blood Transfusion). [file Image_6.pdf]

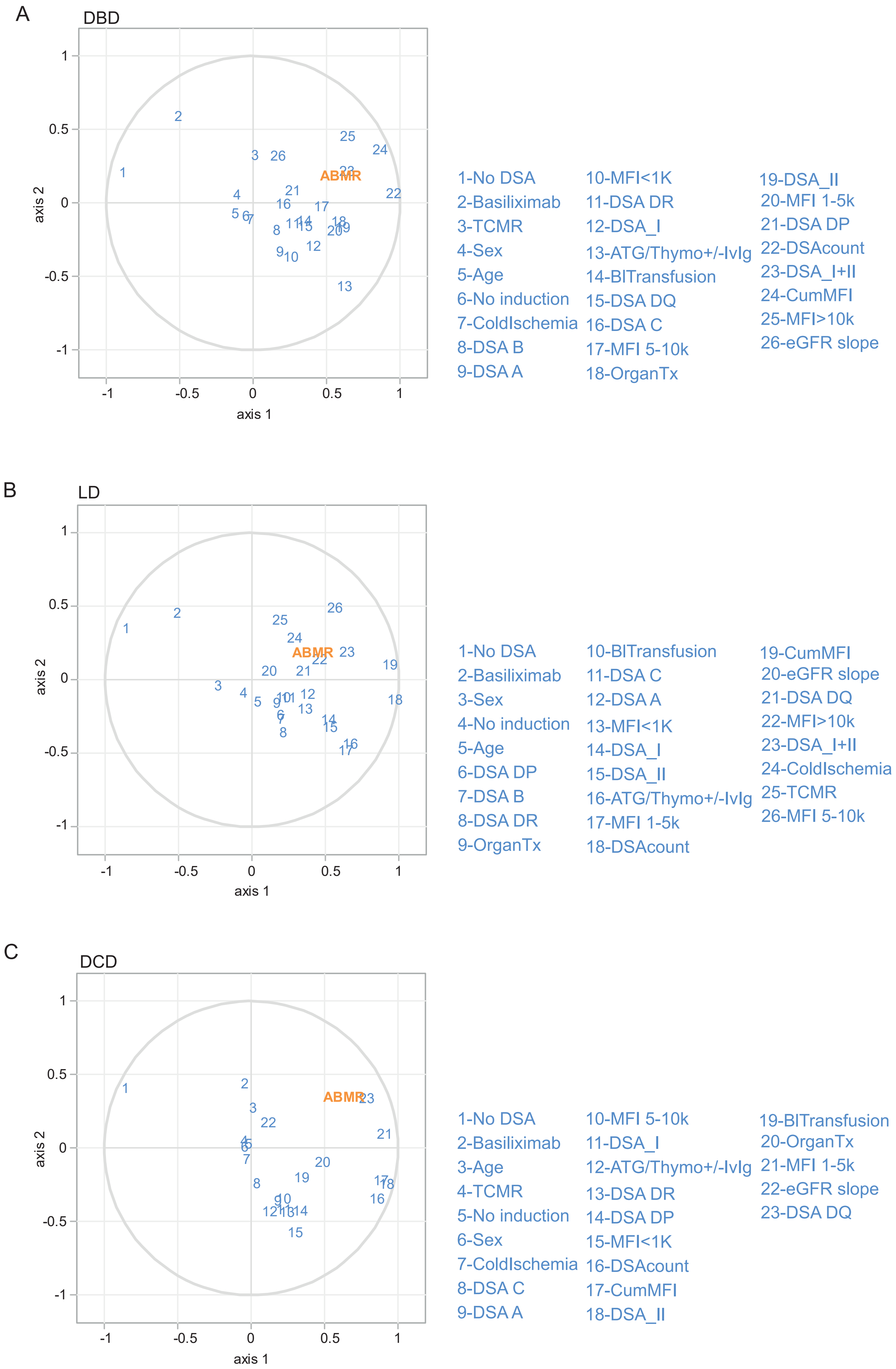

A
DBD
axis 1
axis 2
ABMR
1-No DSA
2-Basiliximab
3-TCMR
4-Sex
5-Age
6-No induction
7-ColdIschemia
8-DSA B
9-DSA A
10-MFI<1K
11-DSA DR
12-DSA_I
13-ATG/Thymo+/-IvIg
14-BlTransfusion
15-DSA DQ
16-DSA C
17-MFI 5-10k
18-OrganTx
19-DSA_II
20-MFI 1-5k
21-DSA DP
22-DSAcount
23-DSA_I+II
24-CumMFI
25-MFI>10k
26-eGFR slope
B
LD
axis 1
axis 2
ABMR
1-No DSA
2-Basiliximab
3-Sex
4-No induction
5-Age
6-DSA DP
7-DSA B
8-DSA DR
9-OrganTx
10-BlTransfusion
11-DSA C
12-DSA A
13-MFI<1K
14-DSA_I
15-DSA_II
16-ATG/Thymo+/-IvIg
17-MFI 1-5k
18-DSAcount
19-CumMFI
20-eGFR slope
21-DSA DQ
22-MFI>10k
23-DSA_I+II
24-ColdIschemia
25-TCMR
26-MFI 5-10k
C
DCD
axis 1
axis 2
ABMR
1-No DSA
2-Basiliximab
3-Age
4-TCMR
5-No induction
6-Sex
7-ColdIschemia
8-DSA C
9-DSA A
10-MFI 5-10k
11-DSA_I
12-ATG/Thymo+/-IvIg
13-DSA DR
14-DSA DP
15-MFI<1K
16-DSAcount
17-CumMFI
18-DSA_II
19-BlTransfusion
20-OrganTx
21-MFI 1-5k
22-eGFR slope
23-DSA DQ
