# Supplementary material for: Donation type and the effect of pre-transplant donor specific antibodies – Data from the Swiss Transplant Cohort Study
Source: Front Immunol. 2023 Feb 15;14:1104371. doi: 10.3389/fimmu.2023.1104371 (PMC9974644; doi:10.3389/fimmu.2023.1104371)
Supplement: Supplementary file 7 [file Table_1.pdf]

Supplementary Table 1. Characteristics of the recipient transplanted with kidney from different donor types

Parameter	DBD total patients (n=1282)	LD total patients (n=803)	DCD total patients (n=130)	p value (DBD vs. LD)	p value (DBD vs. DCD)	p value (LD vs. DCD)	p value (DBD vs. LD vs. DCD)
Recipient age (mean value)	54.80	49.15	53.15	<0.001	0.932	0.03	<0.001
Recipient (female) gender	38%	34%	41%	<0.001	0.011	0.196	0.137
Donor age (mean value)	51.53	53.70	50.88	<0.001	<0.001	0.004	0.008
Donor (female) gender	44%	53%	38%	<0.001	<0.001	0.504	<0.001
Mean follow-up time (year)	5.81	6.47	4.34	0.557	<0.001	<0.001	<0.001
Cold ischemia time (DD)/h (mean value)	8.59	4.76	7.00	0.742	0.555	0.464	<0.001
HLA mismatches							
HLA A (yes)	14%	18%	7%	<0.001	<0.001	<0.001	0.003
HLA B (yes)	7%	13%	5%	<0.001	0.091	<0.001	<0.001
HLA DRB1 (yes)	16%	17%	14%	0.458	0.094	0.175	0.710
Induction therapy (yes)	98%	97%	100%	0.012	<0.001	<0.001	0.067
